# Proteomics identification of radiation-induced changes of membrane proteins in the rat model of arteriovenous malformation in pursuit of targets for brain AVM molecular therapy

**DOI:** 10.1186/s12014-018-9217-x

**Published:** 2018-12-26

**Authors:** Margaret Simonian, Dyna Shirasaki, Vivienne S. Lee, David Bervini, Michael Grace, Rachel R. Ogorzalek Loo, Joseph A. Loo, Mark P. Molloy, Marcus A. Stoodley

**Affiliations:** 1 Department of Clinical Medicine, Faculty of Medicine and Health Sciences, Macquarie University, Sydney, NSW Australia; 2 Department of Biological Chemistry, David Geffen School of Medicine, University of California Los Angeles (UCLA), 611 Charles E. Young Drive East, Los Angeles, CA 90095 USA; 3 Neurosurgery Department, Bern University Hospital, Bern, Switzerland; 4 Genesis Cancer Care, Macquarie University Hospital, Sydney, NSW Australia; 5 Department of Chemistry and Bimolecular Sciences, Australian Proteome Analysis Facility (APAF), Macquarie University, Sydney, NSW Australia; 6 Lawrence Penn Chair of Bowel Cancer Research, Faculty of Medicine and Health, Northern Clinical School, Sydney, Australia

**Keywords:** In vivo biotinylation, AVM animal model, Membrane proteins, Radiosurgery

## Abstract

**Background:**

Rapid identification of novel targets and advancement of a vascular targeting strategy requires a comprehensive assessment of AVM endothelial membrane protein changes in response to irradiation. The aim of this study is to provide additional potential target protein molecules for evaluation in animal trials to promote intravascular thrombosis in AVM vessels post radiosurgery.

**Methods:**

We employed in vivo biotinylation methodology that we developed, to label membrane proteins in the rat model of AVM post radiosurgery. Mass spectrometry expression (MS^E^) analysis was used to identify and quantify surface protein expression between irradiated and non irradiated rats, which mimics a radiosurgical treatment approach.

**Results:**

Our proteomics data revealed differentially expressed membrane proteins between irradiated and non irradiated rats, e.g. profilin-1, ESM-1, ion channel proteins, annexin A2 and lumican.

**Conclusion:**

This work provides additional potential target protein molecules for evaluation in animal trials to promote intravascular thrombosis in AVM vessels post radiosurgery.

**Electronic supplementary material:**

The online version of this article (10.1186/s12014-018-9217-x) contains supplementary material, which is available to authorized users.

## Introduction

Brain AVMs are the major cause of hemorrhagic stroke in adults and young children with each haemorrhage caring a 50% risk of death or morbidity [1]. Radiosurgery is the treatment recommended for lesions < 3 cm in diameter. Radiosurgery is relatively safe when treating small lesions < 2.6 cm in diameter, without the risk of high radiation exposure to the surrounding tissues. Vascular occlusion after radiosurgery may take 2–3 years to complete therefore the risk of haemorrhage is not eliminated until complete obliteration is achieved [2, 3]. Promoting rapid thrombosis after radiosurgery by targeting endothelial surface discriminating proteins has the potential to overcome the downside of radiosurgery. The processes of AVM vessel occlusion after radiosurgery are not understood completely, it is suggested to be involved a combination of cellular proliferation and intravascular thrombosis [4, 5]. We have developed an animal model of AVM and demonstrated the ability of stimulating thrombosis in AVM vessels after radiosurgery by non-ligand vascular targeting approach, however only small vessels showed a sign of thrombosis, and the non-ligand injection is not safe for use in humans [6–8]. Therefore a ligand based approach has the potential to overcome these obstacles. To achieve this goal, we have developed in vitro and in vivo biotinylation perfusion methodology to label endothelial cell surface proteins in murine bEnd. 3 cell cultures and the animal model of AVM [9, 10] and we have shown the feasibility of employing proteomics methods on the arteriovenous fistula (AVF) tissues harvested from the animal model [9, 10]. Here, we employ our in vivo methodology to label, then identify and quantify the differentially expressed membrane proteins in the irradiated and non irradiated animals using a label-free quantitative mass spectrometry of expression (MS^E^) technique.

Based on our previous proteomics study of the murine endothelial cell cultures (bEnd.3), the most extensive membrane protein changes in response to irradiation were observed at 24 h post irradiation [11]. Therefore in this study, the membrane protein changes in the rat model of AVM were studied at 24 h post irradiation. Up-regulated membrane proteins in the irradiated rats compare to the controls will be investigated as potential targets for the AVM ligand-directed molecular therapy to stimulate rapid thrombosis in AVM vessels post radiosurgery.

## Materials and methods

### Rats

An arteriovenous fistula (AVF) was created in six *Sprague*-*Dawley* male rats weighing from 360 to 411 g, according to Yassari et al. protocol [6]. Rats were returned to the animal care facility after the operation and monitored daily for the first week then weekly for another 5 weeks, allowing the fistula to reach maturity.

### Radiosurgery of the rats

After 6 weeks of creating the fistulas, three rats were irradiated using the Leksell Gamma knife (Elekta, Stockholm, Sweden) at Macquarie University Hospital by delivering 15 Gy to the AVF tissues directly, minimizing the dose to the trachea to less than 10 Gy. The remaining three rats were used as controls.

### In vivo biotinylation perfusion

After 24 h of irradiation, all six rats were narcotized, prepared and perfused per Simonian et al. [9] protocol, with a slight modification. A Gilson Minipuls 3 perfusion pump attached to a tube and needle was used to perfuse the rats with 1 mL of saline (NaCl) with a flow rate of 50 mL/min to wash away the blood, immediately followed with 100 mL of freshly prepared biotinylation solusion [1.5 mg/mL of Sulfo-NHS-LC-Biotin in pre warmed PBS at 37 °C + 10% Dextran 40] by pressing the syringe plug with a flow rate of 25 mL/min while monitoring the pressure and keeping it constant at ~ 100 mm Hg. After 5 min of perfusing the biotinylation solution, the rats were injected with 100 mL of (50 mM Tris–Hcl in PBS + 10% Dextran 40) with a flow rate of 30 mL/min to wash out excessive biotinylation reagent, then were perfused with 200 mL saline at 30 mL/min to wash away Dextran. The fistula tissues were then excised and the surrounding fat and muscle tissue were removed. The vascular tissues were placed in a 1 mL Eppendorf tube and transferred to a − 80 °C freezer immediately.

### Membrane protein extraction

The harvested vascular tissues were homogenized using tissue grinder with pestle (Wheaton) in 1 mL of lysis buffer (20 mM HEPES, 150 mM NaCl, 10 mM NaF, 1 mM Na-EDTA, 1 mM Na-EGTA, pH 7.5, pH adjusted with NaOH, 0.1% Triton-X v/v) + protease inhibitor (4 µL per mL of HEPES buffer, Sigma P-2714). Samples were probe sonicated for 15 s, three times using the Branson Sonifier 450 (John Morris Scientific) and centrifuged in a pre-cooled rotor at 1500×*g* for 15 min at (4 °C). Supernatants were collected and pellets were re-lysed with 0.5 mL of HEPES buffer (same as above steps). Supernatants were collected and pooled with previous supernatants, the final volume of supernatants were ~ 1.5 mL. Sodium Bicarbonate solution (0.1 M, pH 11) was added to pooled supernatants (up to 5 mL) and incubated for 1 h at 4 °C on rocking platform. After incubation, samples were centrifuged at 100,000×*g* for 45 min at 4 °C. Pellets were washed with 0.5 mL of 100% cold acetone twice and left to dry. Pellets were then dissolved with 200 µL of 100 mM Amonium Bicarbonate containing 10 mM DTT (freshly prepared) in water bath sonication (FS30H, Fisher Scientific) for 20 min and then incubated for 1 h at 37 °C to reduce the samples. To alkalize the samples, 5µL of 1 M idoacetamide stock was added to make final concentration to 20 mM idoacetamide and incubate in dark at room temperature for 30 min. Samples volume were then brought up to 5 mL with 100 mM Amonium Bicarbonate and centrifuged at 100,000×*g* for 1 h at 4 °C. Pellets were dissolved with 400 µL of 100 mM Amonium Bicarbonate in water bath sonication then 600 µL of methanol was added.

### Capture of biotinylated proteins

Biotinylated proteins were captured on streptavidin Sepharose (GE health care, USA). Five hundred microlitres of streptavidin Sepharose were washed three times with buffer A containing (1% w/v NP40, 0.5% w/v SDS in PBS), then three times with 500 µL PBS. Samples then were incubated with washed streptavidin Sepharose for 2 h at room temperature with gentle rotation. Streptavidin Sepharose was pelleted by centrifugation at 1600×*g* for 5 min. Unbound proteins were eliminated by washing three times with 1 mL of 1% Triton-x (v/v), once with 400 µL of 1% SDS (w/v) and five times with 1 mL of digestion buffer 0.25 mM ammonium bicarbonate. The use of high salt concentration and NP40 detergent in the washing buffers minimises the non-specific interactions of biotin and streptavidin.

### Tryptic digestion of biotinylated proteins and MS^E^ analysis

Streptavidin Sepharose was re-suspended in 200 µL of digestion buffer. Twenty microlitres of trypsin were then added and incubated overnight at 37° C. The samples were centrifuged at 14,100×*g* for 2 min at room temperature and the supernatant was collected. Chromatographic separation was achieved using Waters UPLC column (1.7 µm, 75 µm × 150 mm, 10 K psi). The mobile phase, used at a flow rate of 0.3 µL/min, with a gradient of a mixture of (A) 0.1% formic acid in water and (B) 0.1% formic acid in acetonitrile programmed as follows: initial 97% A for 1 min, reduced to 60% A in 60 min, then decreased to 5% in 2 min, held for 15 min, again increased to 97% A in 3 min. The column temperature was set at 28 °C.

Mass spectrometry analysis was performed utilizing a Waters Xevo quadrupole time of flight (Q-TOF) micro™ mass spectrometer coupled directly to Waters nanoACQUITY UPLC system (Waters Corp). Peptides were separated with a UPLC BEH C18 Column (1.7 µm, 75 µm × 150 mm, 10 K psi). The mobile phase, used at a flow rate of 0.3 µL/min, with a gradient of a mixture of (A) 0.1% formic acid in water and (B) 0.1% formic acid in acetonitrile was programmed as follows: initial 97% A for 1 min, decreased to 60% A in 60 min, then decreased to 5% for 2 min, held at this for 15 min, again increased to 97% A in 3 min. The column temperature was set at 28 °C.

All analysis was performed using positive mode electrospray ionization (ESI). The LC–MS spectrometer was operated in the MS^E^ data independent acquisition mode. LC–MS data was collected in an alternating low energy MS and elevated energy MS/MS (MS^E^) mode of acquisition. In low energy MS mode the data were collected at a constant collision energy of 6 eV. In elevated energy MS/MS mode the collision energy was ramped from 15 to 40 eV on laboratory frame energy to collect product ions of all precursors identified from the MS scan. Samples were injected into the mass spectrometer in triplicates.

### Data analysis

The LC MS and LC MS/MS data were processed using ProteinLynx Global Server (PLGS) version 2.5 (Waters Corporation). The quantification of protein levels was achieved by the addition of an internal protein standard (alcohol dehydrogenase trypsin digest) to which the data set was normalized. The protein identification was based on MS/MS peak lists which were generated by MS^E^ data independent collision induced fragmentation using a *Rattus* database. Protein identifications were accepted with greater than three fragment ions per peptide, seven fragment ions per protein and one unique peptide per protein identified. Carbamidomethyl cysteine was set as a fixed modification while oxidized methionine was set as a variable modification. Trypsin was set as a proteolytic enzyme, and up to two missed cleavages were allowed. Peptide tolerance set at 10 ppm with fragment ion tolerance of 0.5 amu. When a peptide was not detected in the MSE experiment a nominal amount of 0.01 was reported to avoid zeros in subsequent calculations. The *p* values of the expression ratios of irradiated and control rats were determined using two tailed student’s t test.

## Results

To determine the effect of irradiation on AVF endothelium, and to study the up-regulated membrane proteins in response to irradiation, 18 mass spectrometry runs were analysed from 3 irradiated rats and 3 control rats. Triplicate samples were run for each rat. The proteomics data detected a total of 74 proteins in the irradiated rats, 20 of them were annotated as membrane proteins. A total of 104 proteins were detected in control rats, 37 of them were annotated as membrane proteins.

Twelve membrane proteins shared their presence in both irradiated and control rats (Table 1). Their average concentrations (fmol) and average ratios of expression (irradiated: control) were calculated to determine the changes in membrane protein level following irradiation.

**Table 1:** Membrane proteins shared between irradiated (R) and control (C) rats, their sequence accession number, molecular weight, average concentration on column (fmol) and average concentration ratios (irradiated: control); n = 18

Protein name	Accession #	MW	Ave (fmol) in R	SD	Ave (fmol) in C	SD	Fold change (R:C)	(*p*)
Alpha 1 macroglobulin	Q63041	168,494.1	4.2	1.67	5.8	0.29	0.8	0.222
Biglycan	P47853	42,105.52	1.5	0.1	3.0	0.26	0.5	0.32
Annexin A1	P07150	39,171.71	1.4	0.83	2.1	0.13	0.7	0.288
Annexin A2	Q07936	38,963.45	5.0	0.07	6.0	0.60	0.8	*0.025*
Lumican	P51886	38,678.25	2.6	0.30	4.1	0.18	0.6	*0.041*
GTPase	Q8K3L6	37,665.02	5.2	0.2	18.9	0.1	0.3	
Prolargin	Q9EQP5	43,521.63	3.5	0.62	3.9	2.28	0.9	0.727
Serine protease inhibitor	P05544	46,448.34	1.1	0.01	8.1	0.01	0.1	0.82
Alpha 1 inhibitor 3	P14046	165,142.2	6.2	0.32	2.7	0.05	2.3	0.164
Collagen alpha 1	P02454	138,980.1	7.4	0.40	7.3	0.05	1.0	0.618
Collagen alpha 2	P02466	130,077.4	2.1	0.20	7.8	0.21	0.3	0.07
Decorin	Q01129	40,147.32	1.8	1.15	3.1	0.06	0.6	0.205

*p* < 0.05 indicate statistical significance

The expression of Alpha-1 inhibitor protein increased in irradiated rats by (2.3) fold, however this increase wasn’t significant (*p *= 0.164), while annexin-A2 and lumican showed significant decrease (*p* = 0.025 and 0.041 respectively).

Interestingly, eight membrane proteins were detected in irradiated rats only, and not in control rats, due to their extremely low concentrations in the control rats (Table 2), such as profilin-1, ESAM-1, potassium voltage gated channel protein and chloride intracellular channel protein-2, they have shown significant up-regulation (293 fold, 28 fold, 390 fold and 60 fold respectively).

**Table 2:** Membrane proteins present in irradiated rats (R) only, their accession number, molecular weight average concentration on column (fmol), and average concentration ratios (irradiated: control); n = 9

Protein name	Accession #	MW	Ave (fmol) R	SD	Ave fmol C	Fold R:C	(*p)*
Profilin 1	P62963	15,128.34	2.9	3.27	0.01	293.625	*0.001*
Endothelial cell specific molecule-1	P97682	21,101.27	0.6	0.39	0.01	28.98	*0.002*
Bone morphogenetic protein 3	P49002	53,416.56	7.9	4.95	0.01	790	*0.001*
Potassium voltage gated channel subfamily A member 5	P19024	67,237.32	3.4	2.42	0.01	340	*0.005*
Myelin protein	P06907	27,741.78	7.4	6.48	0.01	737.5	*0.017*
Chloride intracellular channel protein 2	Q5M883	28,446.33	0.6	0.39	0.01	60	*0.051*
Vomeromodulin	Q63751	10,890.35	7.2	5.07	0.01	720	0.090
Prothyroliberin	P01150	29,454.97	5.3	3.70	0.01	530	0.022

*p* < 0.05 indicate statistical significance

A list of all membrane proteins that were present in irradiated rats are presented in (Table 3), while the membrane proteins that were present in the control rats are presented in (Table 4).

**Table 3:** Membrane proteins identified by LC–MS/MS in irradiated rats, their accession number and classifications

Protein name	Uniprot accession #	Membrane protein classification
Profilin-1	P62962	Cytoskeleton
Annexin A2	P07356	Extracellular matrix
Decorin	Q01129	=
Lumican	P51886	=
Biglycan	P47853	=
Annexin A1	P10107	=
Collagen alpha 1	P02454	=
Prolargin	Q9EQP5	=
Collagen alpha 2	P08123	=
Alpha 1 macroglobulin	P06238	=
Prothyroliberin	P01150	=
GTPase	P20171	Lipid anchor
Alpha 1 inhibitor 3	P04585	=
Mylein	P02688	Peripheral
Potassium voltage gated channel	P15387	Multi pass
Serine protease inhibitor	P27958	Single pass type 1
Endothelial cell specific molecule 1	P35918	=
Chloride intracellular channel protein 2	O35433	Multi pass
Vomeromodulin	Q63751	Extracellular matrix
Bone morphogenetic protein 3	Q06826	=

**Table 4:** Membrane proteins identified my LCMS/MS in control rats, their accession number and classifications

Protein name	Uniprot accession #	Membrane protein classification	Protein name	Uniprot accession #	Membrane protein classification
Annexin A2	P07356	Extracellular matrix	GTPase	P20171	Lipid anchor
Annexin A1	P10107	=	Serine protease inhibitor	P27958	Single pass type 1
Collagen alpha 1	P02454	=	Alpha 1 inhibitor3	P10824	Peripheral
Lumican	P51886	=	Annexin 5	P14668	Extracellular matrix
Collagen alpha 2	P08123	=	Vesicle associated memb.prot.3	P63035	Single pass type IV
Neuromodulin	Q63751	=	Fibroblast growth factor 16	P13109	Extracellular matrix
Decorin	Q01129	=	Prolargin	Q9EQP5	=
Regulating synaptic membrane exocytosis protein	Q9JIR4	Peripheral	Biglycan	P47853	=
Ig gamma 2A chain	P01865	Single pass	Alpha 1 macroglobulin	P06238	=
ATP synthase subunit alpha	P10719	Peripheral	Alpha actinin 4	P57780	=
Serotransferrin	P12346	Basement	Jouberin	Q6DTM3	=
Heat shock 75	P48721	Extracellular	Beta defensin 15	Q322H6	=
PKHD domain containing transmembrane protein C1	Q6T3A5	Transmembrane	Plasma kallikrein	P14272	=

Non membrane protein expression also differed between irradiated and control rats. Actin, myosin and tubulin were highly expressed in irradiated rats, while vimentin and fibroblast growth factor-16 expression were detected in control rats only (Additional file 1: Tables S1 and S2, Additional file 2: Table S4).

## Discussion

This study aimed to identify differentially expressed surface proteins in the animal model of AVM subjected to irradiation, as these proteins could have utility for molecular targeting. In general the total number of proteins detected by mass spectrometry analysis in the control rats was higher than irradiated rats. This is expected since irradiation at doses 15–20 Gy may cause some cell death while doses higher than 100 Gy causes cell hypertrophy [12].

The eight membrane proteins that were present mainly in irradiated rats, such as profilin-1, ESAM-1, potassium voltage gated channel and chloride intracellular channel protein 2, are of importance to this study due to their significant up-regulation (293 fold, 28 fold, 390 fold and 60 fold respectively), as well as the non-membrane proteins highly expression in irradiated rats, such as actin, myosin and tubulin.

Profilin-1 is an actin binding protein; hence its increased expression is expected due to increase expression of actin in irradiated rats. Prifilin-1 belongs to the profilin family, at high concentrations; it increases the ADP-to-ATP exchange on G-actin, and prevents the polymerization of actin, whereas at low concentrations it enhances it [13, 14]. Profilin-1 contribution to actin dynamics at plasma membrane is part of many other activities. Profilin-1 has been regarded as a tumour-suppressor molecule for breast cancer and the up-regulation of prifilin-1 after irradiation, induced apoptosis in pancreatic cancer cells [14, 15]. A ligand for profilin-1 have been identified, p42^*pop*^ which is a novel Myb-transcription factor [16]. Due to its association with plasma membrane actins, profilin-1 may well be regarded as a potential candidate for the AVM ligand directed vascular therapy to deliver thrombotic agents.

Potassium voltage gated channel protein is another membrane protein that was up-regulated significantly in irradiated rats. This protein is an ion channel transport protein and membrane potential that facilitates the flow of potassium ions down an electrochemical gradient (Zhang et al. 2003). In the brain the greatest density occurs in the cerebral cortex. Previous studies have shown increased expression of this protein in response to irradiation [17, 18]. Potassium channels are commonly expressed in tumour cells and have been a target for many drugs. Different agents have been used to target potassium channels in animal tumour models and in clinical trials; they have also been used for the treatment of other diseases such as type 2 diabetes and hypertension, with minimal side effects [19–21].The interference with potassium channels offered a new therapeutic characteristic for cancer treatment because this channel is sometimes favourably expressed in tumour cells and sometimes the abnormally expressed form is different from the physiological one, this made it easy to block the potential side effects of the drug by channel targeting in ordinary tissues [18, 22]. Our future work will focus on studying the differential expression of potassium voltage gated channels in AVM vessels and normal vessels. The fact that this protein is extracellularly accessible may simplify targeting and drug design. This protein will be investigated as a potential target for ligand directed therapy for brain AVMs.

Chloride intracellular channel protein 2 is another voltage gated ion channel transmembrane protein that was up-regulated significantly in irradiated rats. Chloride channel proteins have also been used as molecular targets for cancer therapy [23]. Previous studies on human lung cancer cells and laryngeal cancer cells have shown increased expression of chloride intracellular channel proteins 4 and 1 in response to irradiation, suggesting these proteins as important and novel targets for anti cancer therapy and radiotherapy for cancer cells [24, 25]. Therefore our future work will also include extensive study of chloride intracellular channel protein 2 as potential target for molecular therapy for brain AVM.

Endothelial cell specific molecule-1 (ESM-1) is yet another up-regulated membrane protein expressed significantly in the irradiated rats. ESM-1 is endothelial cell adhesion molecule that is also expressed on platelets. In 2009, Stalker et al., showed that after platelet activation, ESM-1 was localized to the junctions between adjacent platelets, suggesting a role for this protein in thrombus formation [26]. Exposed tissue factor that was found in some irradiated blood vessels lacking the endothelial lining suggested one mechanism in which thrombosis may occur post radiosurgery, however no significant differences in expression have been shown after irradiation [7, 8]. Further investigation of the ESM-1 role in thrombosis of AVM vessels may provide potential molecular target for the AVM vascular therapy.

A number of non-membrane proteins also showed increased expression in the irradiated rats, such as actin, myosin and tubulin. This data is in agreement with our in vitro proteomics study of the murine cerebral endothelial cells in response to irradiation [11]. The expression of these cytoplasmic proteins may well be the result of radiation-stimulated surface expression, as we have used Sulfo-NHS-LC-biotin in this study, which is known to inhibit cell membrane penetration [27]. Previous in vitro studies of endothelial cells have shown cell surface translocation of intracellular proteins in response to irradiation [11, 28, 29]. Additionally, this data also in accordance with previous data obtained from human cerebral endothelial cultures where it was suggested that radiosurgery by Gamma knife induces transformation of fibroblasts into contractile elements (i.e. actin and myosin) in AVM vessels similar to myofibroblasts, which may contribute to the obliteration of AVMs after radiosurgery [30, 31]. Furthermore, in 2009 a study by Sekis et al., showed that irradiation did not up-regulate the vascular endothelial growth factor in masctocytoma cell lines [32]. Interestingly in this study, fibroblast growth factor was detected mainly in control rats AVF tissues; this may support the transformation of fibroblast into contractile elements in the irradiated rats. The expression patterns of these proteins are intriguing; they could potentially be targeted with the use of ligands to deliver thrombotic agents to AVM vessels.

Our previous proteomics investigation of the murine endothelial cerebral cell cultures (bEnd3) revealed a large number of membrane proteins that were differentially expressed between irradiated and non-irradiated cells [11], many of those proteins are identified in this study as well ((Additional file 1: Table S3)), this consistency is important in candidate selection for AVM molecular therapy. The proteins identified from our in vitro and in vivo proteomics studies, are currently being investigated as potential targets for the ligand-directed molecular targeting trials in the rat model of AVM. Future studies will further include irradiation-induced changes in human primary endothelial cell cultures from resected AVM tissues. The significance of this novel research lies in its potential for rapid translation into therapies for currently untreatable brain AVMs, and for patients who currently have to wait up to 3 years after undergoing radiosurgery, for their AVM to be occluded completely, while they remain at risk of haemorrhage.

## Additional files

**Additional file 1: Table S1.** Expression of actins, tubulin, myosin, fibroblast growth factor-16 and vimentin in control rats. **Table S2**. Expression of actins, tubulin and myosin in irradiated rats. **Table S3**. Membrane proteins present in murine endothelial cell cultures, and the rats model of AVM.

**Additional file 2: Table S4.** MSE raw data for one control rat, as an exemplification.
