# Supplementary material for: Proteomics identification of radiation-induced changes of membrane proteins in the rat model of arteriovenous malformation in pursuit of targets for brain AVM molecular therapy
Source: Clin Proteomics. 2018 Dec 26;15:43. doi: 10.1186/s12014-018-9217-x (PMC6305998; doi:10.1186/s12014-018-9217-x)
Supplement: Supplementary file 1 — Additional file 1: Table S1. Expression of actins, tubulin, myosin, fibroblast growth factor-16 and vimentin in control rats. Table S2. Expression of actins, tubulin and myosin in irradiated rats. Table S3. Membrane proteins present in murine endothelial cell cultures, and the rats model of AVM. [file 12014_2018_9217_MOESM1_ESM.docx]

**Additional file 1**

**Table S1.** Expression of actins, tubulin, myosin, fibroblast growth factor-16 and vimentin in control rats

|  | **Accession #** | **Protein name** | **Replication** | **Ave matched Peptides** | **Ave (Fmol)** | **Ave Seq. Coverage** | **Ave score** |
| --- | --- | --- | --- | --- | --- | --- | --- |
|  | P60711 | Actin cytoplasmic 1 | 3 | 12 | 10.7651 | 36.17667 | 1613.145 |
| Rat 1 | P63269 | Actin gamma enteric | 1 | 13 | 20.148 | 30.055 | 1843.409 |
|  | P68370 | Tubulin alpha 1A | 3 | 5.3 | 3.1273 | 11.163 | 375.8343 |
|  | P12847 | Myosin 3 | 1 | 32 | 0.4095 | 1.657 | 40.9358 |
|  |  |  |  |  |  |  |  |
|  | P68370 | Tubulin alpha 1A | 3 | 5.3 | 2.540633 | 10.56667 | 558.2356 |
| Rat 2 | P60711 | Actin cytoplasmic 1 | 2 | 12.5 | 8.93525 | 32.265 | 1397.233 |
|  | P63269 | Actin gamma enteric | 3 | 11 | 17.24823 | 28.63333 | 1762.532 |
|  | O54769 | Fibroblast growth factor 16 | 2 | 7 | 74.3576 | 20.765 | 1453.23 |
|  |  |  |  |  |  |  |  |
|  | P31000 | Vimentin | 3 | 14.33 | 2.718367 | 16.23667 | 147.9262 |
| Rat 3 | P60711 | Actin cytoplasmic 1 | 3 | 14 | 8.3632 | 35.37667 | 1336.817 |
|  | P63269 | Actin gamma enteric | 2 | 12.5 | 18.5424 | 29.52 | 1640.717 |
|  | P68370 | Tubulin alpha 1A | 1 | 7 | 1.5156 | 9.315 | 98.3372 |
|  | P04462 | myosin 8 | 2 | 5 | 0.42985 | 7.276 | 88.84755 |
|  | O54769 | Fibroblast growth factor 16 | 1 | 4 | 36.2498 | 7.865 | 85.3561 |

**Table S2.** Expression of actins, tubulin and myosin in irradiated rats

|  | **Accession #** | **Protein name** | **Replication** | **Ave matched Peptides** | **Ave (Fmol)** | **Ave Seq. Coverage** | **Ave score** |
| --- | --- | --- | --- | --- | --- | --- | --- |
|  | P60711 | Actin cytoplasmic 1 | 3 | 7 | 13.181 | 20.265 | 287.2407 |
|  | P63269 | Actin gamma enteric | 2 | 8.5 | 25.272 | 23.935 | 358.1399 |
| Rat 1 | P62738 | Actin aortic smooth muscle | 3 | 9 | 35.507 | 31.03 | 517.838 |
|  | P68370 | Tubulin alpha 1A | 2 | 5 | 5.2278 | 12.195 | 398.9251 |
|  |  |  |  |  |  |  |  |
|  | P60711 | Actin cytoplasmic 1 | 3 | 12 | 10.7651 | 36.17667 | 1613.145 |
| Rat 2 | P62738 | Actin aortic smooth | 2 | 10.5 | 17.905 | 31.565 | 1669.235 |
|  | P68370 | Tubulin alpha 1A | 3 | 5.3 | 3.1273 | 11.16 | 375.8343 |
|  | P63269 | Actin gamma enteric | 3 | 13 | 20.148 | 30.05 | 1843.409 |
|  | P12847 | Myosin 3 | 3 | 32 | 0.7095 | 1.65 | 40.9358 |
|  |  |  |  |  |  |  |  |
| Rat 3 | P62738 | Actin aortic smooth muscle | 3 | 9 | 20.87737 | 27.49667 | 1048.403 |
|  | P60711 | Actin cytoplasmic 1 | 3 | 9 | 8.5851 | 25.77667 | 962.6231 |
|  | P68370 | Tubulin alpha 1A | 2 | 4 | 7.7532 | 10.755 | 221.3986 |

**Table S3**. Membrane proteins present in murine endothelial cell cultures, and the rats model of AVM

| Annexin A2 | Alpha enolase | Vesicle associated membrane protein | Lumican |
| --- | --- | --- | --- |
| Fibroblast growth factor 16 | Gamma enolase | Prelamin | Decorin |
| Transmembrane protein | Creatine kinase B type | ATP synthases subunit beta | Profilin 1 |
| ATP synthase subunit beta | Rab GDP dissociation inhibitor | Caveolin 1 | Myelin |
| Biglycan | Heat shock cognate 71 | Annexin A1 | Serine protease inhibitor |
| Prolargin | PDI | GTPase IMAP family member 5 | Heat shock protein 75 |
| EPCR | Multimerin 2 | Cell surface glycoprotein | MUC18 |

**Table S4.** MSE raw data for control rat, as an example of other samples raw data presentation/calculations
